# Neutralizing antibody and CD8^+^ T cell responses following BA.4/5 bivalent COVID-19 booster vaccination in adults with and without prior exposure to SARS-CoV-2

**DOI:** 10.3389/fimmu.2024.1353353

**Published:** 2024-03-20

**Authors:** Alexander P. Underwood, Christina Sølund, Kivin Jacobsen, Alekxander Binderup, Carlota Fernandez-Antunez, Lotte S. Mikkelsen, Dilek Inekci, Signe Lysemose Villadsen, Jose A. S. Castruita, Mette Pinholt, Ulrik Fahnøe, Santseharay Ramirez, Liselotte Brix, Nina Weis, Jens Bukh

**Affiliations:** ^1^ Copenhagen Hepatitis C Program (CO-HEP), Department of Immunology and Microbiology, Faculty of Health and Medical Sciences, University of Copenhagen, Copenhagen, Denmark; ^2^ Department of Infectious Diseases, Copenhagen University Hospital, Hvidovre, Denmark; ^3^ Immudex ApS, Copenhagen, Denmark; ^4^ Department of Clinical Microbiology, Copenhagen University Hospital, Hvidovre, Denmark; ^5^ Department of Clinical Medicine, Faculty of Health and Medical Sciences, University of Copenhagen, Copenhagen, Denmark

**Keywords:** SARS-CoV-2, COVID-19, vaccination, neutralization, CD8 T cells

## Abstract

As severe acute respiratory coronavirus 2 (SARS-CoV-2) variants continue to emerge, it is important to characterize immune responses against variants which can inform on protection efficacies following booster vaccination. In this study, neutralizing breadth and antigen-specific CD8^+^ T cell responses were analyzed in both infection-naïve and infection-experienced individuals following administration of a booster bivalent Wuhan-Hu-1+BA.4/5 Comirnaty^®^ mRNA vaccine. Significantly higher neutralizing titers were found after this vaccination compared to the pre-third booster vaccination time point. Further, neutralizing breadth to omicron variants, including BA.1, BA.2, BA.5, BQ.1 and XBB.1, was found to be boosted following bivalent vaccination. SARS-CoV-2-specific CD8^+^ T cells were identified, but with no evidence that frequencies were increased following booster vaccinations. Spike protein-specific CD8^+^ T cells were the only responses detected after vaccination and non-spike-specific CD8^+^ T cells were only detected after infection. Both spike-specific and non-spike-specific CD8^+^ T cells were found at much lower frequencies than CD8^+^ T cells specific to cytomegalovirus (CMV), Epstein-Barr virus (EBV) and influenza (Flu). Taken together, these results show that the bivalent Wuhan-Hu-1+BA.4/5 Comirnaty^®^ mRNA vaccine boosted the breadth of neutralization to newer SARS-CoV-2 variants and that vaccination is able to induce spike protein-specific CD8^+^ T cell responses, which are maintained longitudinally.

## 1 Introduction

Coronavirus disease 2019 (COVID-19), caused by severe acute respiratory syndrome coronavirus 2 (SARS-CoV-2), has led to the deaths of millions globally (1). Following the introduction of approved COVID-19 vaccinations at the end of 2020, although protection from infection was not achieved, cases of severe COVID-19, hospitalization and death dropped significantly (1, 2). However, the protection efficacies of the original vaccines were found to diminish due to a combination of waning immunity and the emergence of new SARS-CoV-2 variants capable of evading prior-established immune responses (1, 3, 4). The evolving SARS-CoV-2 variants are generally characterized by mutations in the surface located spike (S) protein, which is the major protein for host cell entry and the major target for neutralizing antibody (nAb) responses. To improve immunity against emerging SARS-CoV-2 variants, administrations of booster vaccinations have been implemented, and vaccine formulas were updated from a monovalent ancestral S protein (Wuhan-Hu-1 variant) to a bivalent formula containing both the ancestral S protein (Wuhan-Hu-1 S mRNA) and an S protein from a more recent variant (omicron BA.1 or omicron BA.4/BA.5 S mRNA). This vaccine regimen was approved by the food and drug administration (FDA), United Kingdom (UK) government, European Medicines Agency (EMA) and therapeutic goods administration (TGA) for use in the United States (US), Canada, UK, Europe, and Australia. The three major vaccine regimes that have been updated include Comirnaty^®^, Spikevax^®^ and Novavax^®^. However, as implementation of these updated vaccine formulas has been relatively slow, newer omicron variants emerged, such as omicron BQ.1 and omicron XBB.1, which have been shown to be highly resistant to neutralizing activity from both therapeutic monoclonal antibodies and sera from convalescent/vaccinated individuals (5–7). Recent studies have shown that nAb titers correlate with protection from developing severe COVID-19 (8, 9), thus, measurement of nAb titers following vaccination and/or infection is of high importance for prediction of protection efficacies.

Like nAb responses, recent evidence has suggested that SARS-CoV-2-specific CD8^+^ T cell responses correlate with protection from developing severe COVID-19 (10). Unlike nAb epitopes, which are greatly affected by the S protein changes found among the different SARS-CoV-2 variants, CD8^+^ T cell epitopes have been found to be more conserved (11, 12). However, while some CD8^+^ T cell epitopes have been proposed to be more immunodominant (12, 13), a large degree of heterogeneity has been found amongst both convalescent and vaccinated individuals. This is mostly due to the heterogeneity of class I major histocompatibility complexes (MHCs), which are responsible for presenting epitopes to CD8^+^ T cells. Each different type of MHC class I molecule is restricted to the type of epitopes it can present, which are linear amino acid sequences usually 8-11 amino acids in length. Using pools of CD8^+^ T cell epitopes, recent studies have shown that S protein-specific CD8^+^ T cells can be induced following COVID-19 vaccination (14–17), albeit to relatively low frequencies. However, it is not completely clear how these frequencies fluctuate with additional antigen exposures (infection or additional vaccination) nor have other studies compared the frequencies of SARS-CoV-2-specific CD8^+^ T cells to other antigen-specific CD8^+^ T cells or negative controls.

In the present study, the breadth of nAb responses in plasma were assessed in individuals with a COVID-19 vaccination history, including some with subsequent SARS-CoV-2 infection, with a particular focus on nAb responses after a fourth Pfizer/BioNTech (Comirnaty^®^) vaccine dose containing the ancestral (Wuhan-Hu-1) and BA.4/BA.5 bivalent formula. In addition, SARS-CoV-2-specific CD8^+^ T cells were analyzed longitudinally at pre-vaccination (and pre-infection) and approximately 1-month (1M) post vaccination for each vaccination (4 doses total). SARS-CoV-2-specific CD8^+^ T cells following vaccinations were split into S protein-specific and non-S protein-specific and compared between infection-naïve individuals and infection-experienced individuals. Lastly, the frequencies of SARS-CoV-2-specific CD8^+^ T cells were compared to a non-specific epitope and to immunodominant epitopes found in cytomegalovirus (CMV), Epstein-Barr virus (EBV) and influenza (Flu).

## 2 Materials and methods

### 2.1 Study cohort

Participants for this study were selected from the clinical, virological and immunological COVID-19 (CVIC) study, which is a prospective cohort of individuals either infected by SARS-CoV-2 or vaccinated against COVID-19, as previously described (18–20). Participants for this study were selected based on whether they had received the bivalent (Wuhan-Hu-1+omicron BA.4/5 S mRNA) Comirnaty^®^ booster vaccine. Any participants that received a Vaxzervria^®^ or Spikevax^®^ vaccination were excluded from this study. Thus, participants in this study received only Comirnaty^®^ vaccinations. Longitudinal follow up included blood collection at pre-vaccination, 1M post-booster vaccination (second dose), 1M-post second booster vaccination (third dose), pre-third booster vaccination and 1M-post third booster vaccination (fourth dose).

### 2.2 Plasma and peripheral blood mononuclear cell isolation

Following blood collection in ethylenediaminetetraacetic acid (EDTA) tubes, blood was centrifuged at 1800 x *g* for 10 min with the brake turned off. Plasma was then collected and stored at -80°C in cryovials until use. The buffy coat containing peripheral blood mononuclear cells (PBMCs) was collected, combined with phosphate buffered saline (PBS) and added to Sepmate tubes (STEMcell Technologies) containing Ficoll (Sigma-Alrich). The tubes were then centrifuged at 1200 x *g* for 10 min with the brake turned off. The supernatant was then poured into a fresh tube and topped up with PBS. The tubes were then centrifuged at 400 x *g* for 6 min and washed once more with PBS. Following this, the cells were resuspended in RPMI media (Gibco) and counted using a Countess II (Thermo Fisher Scientific). An equal part of pre-chilled freezing media (50% RPMI, 30% FBS and 20% dimethyl sulfoxide) was then added and cells were frozen at -80°C in a CoolCell^®^ container (Corning) in cryovials containing a minimum of 5 x10^6^ cells/vial. For long-term storage, the cryovials were moved to -150°C.

### 2.3 Screening for SARS-CoV-2 infection

As reported previously (19), to determine if a participant had been infected, plasma was screened longitudinally for the presence of anti-nucleocapsid (N) antibodies using a EuroImmun semi-quantitative enzyme-linked immunosorbent assay (ELISA) (PerkinElmer, cat#: EI 2606-9601-2 G). All time points were compared to the pre-vaccination time point and a signal/noise ratio of 3.0 or more was considered positive. In addition, participants were required to report on testing positive for SARS-CoV-2 infection via routine diagnostic polymerase chain reaction (PCR) from nasopharyngeal swabs. Subsequently, any participants that became PCR positive had their nasopharyngeal swab sent to the Department of Microbiology, Copenhagen University Hospital, Hvidovre, Denmark, to see if a viral sequence could be recovered. RNA extraction of samples from swabs was done using PentaBase viral nucleotide purification kit (PentaBase A/S). Sequencing libraries were prepared using Midnight-ONT/V3 primers and Rapid Barcoding Sequencing Kit (Oxford Nanopore Technologies) following the manufacturer’s instructions. Sequencing was performed using R9.4.1 flow cells (Oxford Nanopore Technologies) on a GridION (Oxford Nanopore Technologies) using default parameters and high-accuracy base-calling. Reads were processed using MinKNOWs internal epi2me workflow (v.0.3.14) for SARS-CoV-2 with default parameters. Inside the workflow, variants were called using Nextclade (v.1.11.0) and Pangolin (v.4.0.5). All infections that had a variant identified can be found in **Table 1**.

**Table 1:** Summary of participant information for the hybrid group vaccinated against SARS-CoV-2 with subsequent infection.

Patient ID	Age (years)	Sex	Ethnicity	Second booster vaccination (date)	Second booster vaccination time point (days post vaccination)	N-ELISA positive (date)*	PCR confirmation (date)	Sequence obtained (variant and clade)	Pre-third booster vaccination time point (days post infection)	Third booster vaccination (date)	Third booster vaccination time point (days post vaccination)
mRNA-001	75	Male	Caucasian	22 10 2021	41	6 10 2022	7 04 2022	Omicron BA.2 (21L)	182	6 10 2022	49
mRNA-003	46	Male	Caucasian	22 10 2021	42	1 03 2021	None	None	N/A	27 10 2022	36
mRNA-013	49	Female	Caucasian	16 10 2021	44	11 02 2022	11 01 2022	None	N/A	25 10 2022	36
mRNA-018	62	Female	Caucasian	2 11 2021	36	4 10 2022	31 03 2022	None	187	13 10 2022	34
mRNA-019	48	Female	Caucasian	28 10 2021	DNS	4 10 2022	19 02 2022	Omicron BA.2 (21L)	N/A	4 10 2022	58
mRNA-020	52	Female	Caucasian	2 11 2021	30	11 01 2022	None	None	N/A	5 10 2022	40
mRNA-024	49	Female	Caucasian	20 10 2021	35	3 10 2022	27 03 2022	None	190	4 10 2022	41
mRNA-026	60	Male	Caucasian	11 11 2021	26	6 10 2022	2 05 2022	Omicron BA.2 (21L)	157	6 10 2022	25
mRNA-027	48	Female	Caucasian	27 10 2021	40	31 03 2022	21 02 2022	Omicron BA.2 (21L)	226	5 10 2022	43
mRNA-028	60	Female	Caucasian	28 10 2021	41	13 10 2022	None	None	N/A	13 10 2022	39
mRNA-030	33	Female	Caucasian	1 11 2021	28	22 03 2022	15 02 2022	None	N/A	11 10 2022	36
mRNA-035	53	Male	Caucasian	28 10 2021	27	24 02 2022	24 01 2022	None	247	5 10 2022	43
mRNA-036	33	Female	Caucasian	2 11 2021	29	9 02 2022	12 01 2022	None	N/A	25 10 2022	36
mRNA-037	64	Female	Caucasian	22 10 2021	39	30 11 2021	7 02 2022	None	221	5 10 2022	43
mRNA-038	52	Female	Caucasian	11 11 2021	27	31 03 2022	20 02 2022	None	220	7 10 2022	40
mRNA-052	39	Female	Caucasian	25 10 2021	DNS	6 10 2022	5 01 2022	None	N/A	26 10 2022	42
mRNA-053	61	Female	Caucasian	21 10 2021	40	30 11 2021	None	None	N/A	5 10 2022	33
mRNA-054	49	Female	Caucasian	16 10 2021	DNS	11 10 2022	14 02 2022	None	N/A	12 10 2022	35
mRNA-060	57	Female	Caucasian	10 11 2021	20	1 12 2021	None	None	N/A	11 10 2022	36
mRNA-063	48	Male	Caucasian	8 11 2021	37	1 04 2022	30 01 2022	Omicron BA.2 (21L)	N/A	4 10 2022	50
mRNA-067	32	Female	Caucasian	22 10 2021	33	4 06 2021	28 05 2021	Alpha (20I)	N/A	11 10 2022	27
mRNA-071	29	Female	Caucasian	21 10 2021	DNS	21 10 2022	11 01 2022	Omicron BA.1 (21K)	N/A	27 10 2022	36
mRNA-072	48	Female	Caucasian	21 10 2021	43	29 03 2022	14 02 2022	None	N/A	27 10 2022	35
mRNA-079	48	Female	Caucasian	16 10 2021	48	23 02 2022	16 01 2022	Omicron BA.1 (21K)	255	3 10 2022	44
mRNA-093	50	Female	Caucasian	1 11 2021	DNS	10 10 2022	23 06 2022	Omicron BA.5 (22B)	N/A	25 10 2022	39
mRNA-098	32	Male	Caucasian	11 11 2021	34	4 10 2022	None	None	N/A	4 10 2022	51
mRNA-108	46	Male	Caucasian	28 10 2021	49	25 02 2022	15 01 2022	None	271	13 10 2022	47
* *	* *	*26% male*	* *	* *				* *		* *	
*Median (IQR)*	*49 (46-57)*	74% female		*Mean (SD)*	36 (7)			*Mean (SD)*	215 (36)	*Mean (SD)*	39 (7)

DNS, did not show; N/A, not applicable. *N ELISA negative at prior time points.

### 2.4 Ethics

The study was approved by the Regional Ethical Committee (H-20025872) and Data Protection Agency (P-2020-357), respectively, and was conducted in compliance with the Declaration of Helsinki guidelines. All individuals included in this study were 18 years or older and able to read and speak adequate Danish to provide written informed consent. Participants were required to self-report their age, sex, and ethnicity upon enrolment. Study data was collected and managed using research electronic data capture (REDCap) tools hosted at Copenhagen University Hospital, Hvidovre (21).

### 2.5 Neutralization assay

Initially, all participants included in this study were screened for plasma neutralization using a D614G SARS-CoV-2 isolate (DK-AHH1, Genbank accession number MZ049597), as described previously (19, 20, 22). In brief, virus was added to 2-fold serially diluted plasma at a 1:1 ratio and incubated at room temperature. Following 1h incubation, plasma/virus and antibody/virus complexes were then added to Vero E6 cells (RRID: CVCL_0574) seeded the day before (10^4^ cells/well) in quadruplicate. After 48 hours incubation, the cells were fixed and stained. Spots representing virus infected cells were counted and single outliers were removed as previously described (23). The percentage neutralization was calculated as:


$$\%\;Neutralisation=1\;-\left(\frac{Spot\;count}{Average\;spot\;count\;\left\{virus\;only\;and\;healthy\;controls\right\}}\right)\;x\;100$$

Following this initial screening, selected participants were then screened for plasma neutralization to other SARS-CoV-2 isolates including a delta isolate (DK-AHH3, accession number OP271297) (19), an omicron BA.1 isolate (DK-AHH4, accession number OP271296) (19), an omicron BA.2 isolate (DK-AHH5, accession number OP722493) (24), an omicron BA.5 isolate (DK-AHH6, accession number OP722492) (24), an omicron XBB.1.4 isolate (DK-AHH7, accession number OQ843560, described here for the first time) and an omicron BQ.1.1 isolate (DK-AHH8, clade 22E, accession number OQ843561, described here for the first time). All these isolates were grown in Vero E6 cell culture to obtain a viral stock. Viral stocks were then titrated to a multiplicity of infection (MOI) that could completely infect 10^4^ Vero E6 cells without causing cytopathic effect. All neutralization experiments for these isolates were done as described above for DK-AHH1.

### 2.6 HLA typing

Human leukocyte antigen (HLA) typing of subjects was done using a protocol previously developed by others (25). In brief, subject DNA was obtained by lysing one vial of PBMCs using a DNeasy Blood & Tissue kit (Qiagen) according to the manufacturer’s instructions. This DNA was used as a template for PCR amplification of the HLA A allele using the primers and PCR cycling conditions previously described (25). Following PCR, the products were checked via gel agarose electrophoresis and purified using AMPure XP beads (Qiagen) according to the manufacturer’s instructions. Purified products were then pooled, and library preparation was conducted using a NEBNext Ultra II DNA Library Preparation kit (New England Biolabs). Next generation sequencing (NGS) was performed using the MiSeq platform (Illumina). The data was analyzed using Hisat-genotype analysis pipeline, as previously described (26).

### 2.7 Flow cytometry for screening of antigen-specific CD8^+^ T cells

Frozen PBMCs were thawed at room temperature and immediately transferred to PBS supplemented with 5% fetal bovine serum (FBS). The cell suspension was centrifuged at 500 x *g* for 5 min and the cell pellet was resuspended in PBS supplemented with 5% FBS. Cells were then stained in the dark with Fixable Viability Stain 780 (FVS780, BD Biosciences) for 20 min. The cells were then washed twice with PBS supplemented with 5% FBS and resuspended in Brilliant Stain Buffer (BD Biosciences). The cells were split into four aliquots, one for the non-specific negative control Dextramer reagents, one for the CMV, EBV and Flu (CEF) Dextramer reagents, one for the SARS-CoV-2 S protein-specific Dextramer reagents and one for the SARS-CoV-2 non-S protein-specific Dextramer reagents. Dextramer staining was done in the dark for 30 min according to the manufacturer’s instructions. All Dextramer reagents were labelled with the PE fluorophore and the panel of Dextramer reagents can be found in **Supplementary Table 1**. These epitopes were selected from internal and published knowledge on immunodominant epitopes in relation to these specific HLA types (12, 13). Following this, the cells were stained in the dark for 20 min with anti-CD3-AF700 (BD Biosciences), anti-CD14-APC (BD Biosciences), anti-CD19-APC (BD Biosciences), anti-CD4-BUV395 (BD Biosciences) and anti-CD8-FITC (BD Biosciences). The cells were then washed three times with PBS supplemented with 5% FBS and resuspended in PBS supplemented with 5% FBS. Flow cytometry was then performed on a BD LSR Fortessa (5 laser) analyzer (BD Biosciences) using BD FACSDiva™ software version 6.1.3 (BD Biosciences). Downstream analysis of the flow cytometry data was done using FloJo software version 10.8.1 (BD Biosciences).

### 2.8 Statistics

50% inhibitory dilution neutralization titers (ID_50_) of plasma were calculated in GraphPad Prism (version 9.5.1). Each specific statistical test performed is indicated in the text and figure legends. In brief, data was checked for normal distribution by using QQ-plots and assessed using the Shapiro-Wilk test and Kolmogorov-Smirnov tests. Data that was not found to be normally distributed was analyzed using non-parametric tests and corrected for multiple comparisons as indicated. Data that did not pass the normal distribution tests were plotted with the median and 95% confidence interval. All analyses were two tailed and statistical significance was defined as a *p* value less than 0.05.

### 2.9 Role of funders

The funders had no role in study design, data collection and analysis, decision to publish, or preparation of the manuscript. All authors had full access to all the data in the study and had final responsibility for the decision to submit for publication.

## 3 Results

### 3.1 Study participants vaccinated against COVID-19

**Figure 1:**
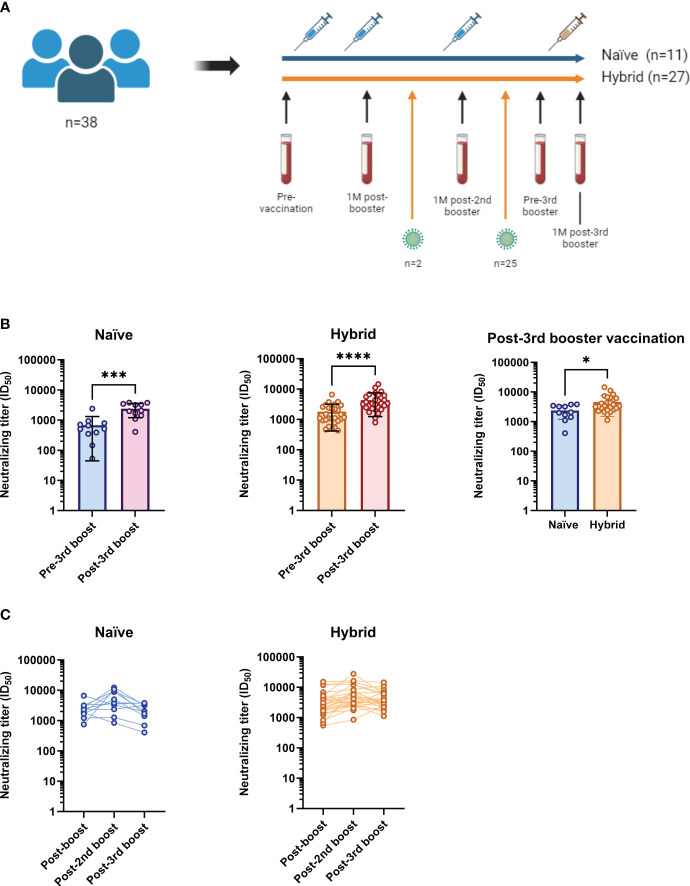
Study cohort summary and examination of neutralizing titers against the ancestral D614G SARS-CoV-2 isolate in the naïve (n=11) and hybrid (n=27) groups following vaccinations against COVID-19. **(A)** Of the 109 participants in the CVIC study cohort, 38 had longitudinal follow-up that included blood collection at 1-month (1M) after bivalent (Wuhan-Hu-1+BA.4/5 S mRNA) third booster vaccination. Collection dates (blood vials) were done at pre-vaccination, 1M post-booster vaccination, 1M post-second booster vaccination, pre-third booster vaccination and 1M post-third booster vaccination. The blue syringes represent vaccination with the monovalent Comirnaty^®^ ancestral formula (Wuhan-Hu-1 S mRNA) and the yellow syringe represents vaccination with the Comirnaty^®^ bivalent (Wuhan-Hu-1+BA.4/5 S mRNA). Eleven of the 38 included did not have a SARS-CoV-2 infection detected throughout follow-up (blue line, termed “naïve”). Twenty seven of the 38 were found to have been infected with SARS-CoV-2 (orange line, termed “hybrid”). Twenty five of the 27 had an infection detected between their second booster and third booster Comirnaty^®^ vaccinations and two had an infection detected between their booster and second booster Comirnaty^®^ vaccinations. **(B)** Comparison of neutralizing titers against the ancestral D614G SARS-CoV-2 isolate between the pre-third booster and post-third booster vaccination time points in the naïve (blue and purple) and hybrid (orange and red) groups. Neutralizing titers were found to be significantly boosted following third booster vaccination in both the naïve (*p*=0.0010, Wilcoxon t test) and hybrid (*p*<0.0001, Wilcoxon t test) groups. When the two groups were compared at the 1M post-third booster vaccination time point (right graph), the hybrid group was found to have significantly higher neutralizing titers (*p*=0.0175, Mann-Whitney U test). **(C)** Longitudinal neutralizing titers at 1M post-booster (post-boost), 1M post-second booster (post-2nd boost) and 1M post-third booster (post-3rd boost) vaccinations in the naïve group (blue) and hybrid group (orange). *p<0.05, ***p<0.001 and ****p<0.0001.

A total of 109 participants vaccinated with a prime-boost Comirnaty^®^ regimen (Wuhan-Hu-1 S mRNA-mRNA) were enrolled in the CVIC study and followed prospectively (18, 19). Among these individuals, 38 received both a third Wuhan-Hu-1 S mRNA Comirnaty^®^ vaccination and a fourth bivalent (Wuhan-Hu-1+BA.4/5 S mRNA) Comirnaty^®^ vaccination and were included in this study. Blood samples collected at pre-vaccination, 1M post-booster vaccination (second dose), 1M post-second booster vaccination (third dose), pre-third booster vaccination and 1M post-third booster vaccination (fourth dose) were analyzed (**Figure 1A**). Twenty-seven of these individuals had had a SARS-CoV-2 infection identified through N protein ELISA, of which 21 were confirmed via routine PCR testing. Twenty five of the 27 had an infection identified between their second booster and third booster Comirnaty^®^ vaccinations and the remaining 2 had an identified infection between their booster and second booster Comirnaty^®^ vaccinations (**Table 1**). Of the 21 with PCR confirmed SARS-CoV-2 infection, 9 had the infecting SARS-CoV-2 variant identified, of which 2 were omicron BA.1, 5 were BA.2, 1 was BA.5 and 1 was the alpha variant (**Table 1**). Regardless of when they were infected, the 27 individuals included in this study with an identified SARS-CoV-2 infection were termed “hybrid” and the remaining 11 that did not have a SARS-CoV-2 infection identified were termed “naïve”. The median age in the hybrid and naïve groups were 49 (interquartile range [IQR]=47-57) and 53 (IQR=48-58), respectively. The percentage of females was 74% in the hybrid group and 82% in the naïve group. A summary of participant information for the hybrid and naïve groups can be found in **Table 1** and **Table 2**, respectively.

**Table 2:** Summary of participant information for the naïve group vaccinated against SARS-CoV-2.

Patient ID	Age (years)	Sex	Ethnicity	Second booster vaccination (date)	Second booster vaccination time point (days post vaccination)	Third booster vaccination (date)	Third booster vaccination time point (days post vaccination)
mRNA-005	41	Female	Caucasian	19 10 2021	36	11 10 2022	38
mRNA-009	55	Female	Caucasian	23 10 2021	33	11 10 2022	35
mRNA-011	48	Female	Caucasian	20 10 2021	40	5 10 2022	34
mRNA-039	53	Female	Caucasian	19 10 2021	51	2 11 2022	33
mRNA-047	73	Male	Caucasian	20 10 2021	51	3 10 2022	44
mRNA-061	63	Female	Caucasian	22 10 2021	39	5 10 2022	34
mRNA-073	50	Female	Caucasian	22 10 2021	38	6 10 2022	42
mRNA-090	56	Female	Caucasian	22 10 2021	39	9 10 2022	33
mRNA-097	52	Female	Caucasian	21 10 2021	34	4 10 2022	42
mRNA-101	25	Male	Caucasian	10 11 2021	20	4 10 2022	45
mRNA-102	58	Female	Caucasian	22 10 2021	DNS	5 10 2022	49
* *	* *	*18% male*	* *				
*Median (IQR)*	*53 (48-58)*	82% female		*Mean (SD)*	38 (9)	*Mean (SD)*	39 (6)

DNS, did not show.

### 3.2 Assessment of neutralizing titers against an ancestral D614G SARS-CoV-2 isolate following serial vaccinations

Firstly, to assess the change in plasma neutralizing titers from the third booster vaccination dose containing the bivalent Wuhan-Hu-1+BA.4/5 S Comirnaty^®^ mRNA, neutralizing titers against an ancestral D614G isolate were assessed in infectious culture systems (20, 22) at pre-third booster vaccination and 1M post-third booster vaccination time points in both naïve and hybrid groups (**Figure 1B**). Neutralization titers (50% inhibitory dilutions [ID_50_s]) were determined by measuring viral infectivity following incubation with serially diluted plasma and comparing to the infectivity at the pre-vaccination time point at a single plasma dilution (1/10). For both the naïve and hybrid groups, significantly higher neutralizing titers were found at the 1M post-third booster vaccination time point when compared to their respective pre-third booster vaccination time point (*p*<0.001, Wilcoxon t tests). Compared to the naïve group at 1M post-third booster vaccination, the hybrid group had significantly higher neutralizing titers (*p*=0.0175, Mann-Whitney U test). Next, neutralizing titers were assessed longitudinally in each group at 1M post-booster, 1M post-second booster and 1M post-third booster vaccination time points (**Figure 1C**). Although both groups appeared to have the highest neutralizing titers against the ancestral D614G isolate at the 1M post-second booster vaccination time point, there were no significant differences found in either group between the time points (*p*>0.05, Kruskal-Wallis tests).

### 3.3 Assessment of neutralizing breadth against delta and omicron SARS-CoV-2 isolates after second and third booster vaccinations

**Figure 2:**
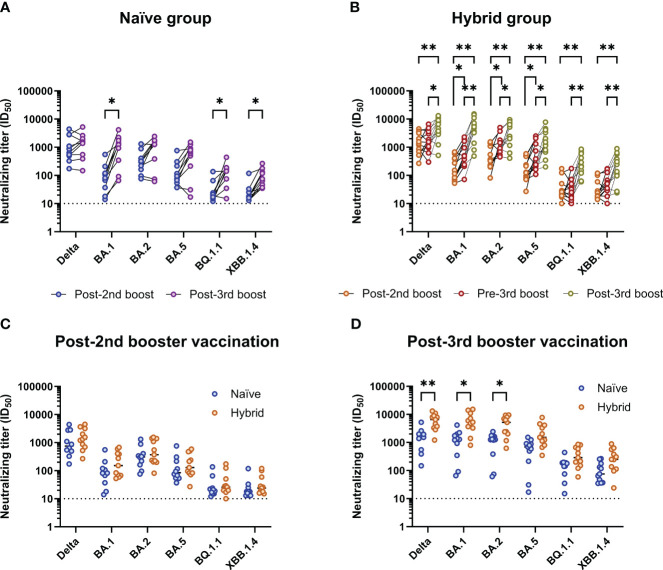
Analysis of breadth of neutralization to different SARS-CoV-2 isolates in the naïve (n=11) and matched hybrid (n=11) groups. **(A)** Comparison of neutralizing titers against different SARS-CoV-2 isolates in the naïve group between the 1M post-second (blue) and 1M post-third booster (purple) vaccination time points. Neutralizing titers against the omicron BA.1, BQ.1.1 and XBB.1.4 isolates were found to be significantly higher at the 1M post-third booster vaccination time point (*p*<0.05, Bonferroni-Dunn corrected multiple Wilcoxon t tests). **(B)** Comparison of neutralizing titers against different SARS-CoV-2 isolates in the hybrid group (infected between the second booster and third booster Comirnaty^®^ vaccinations; matched with naïve group) between the 1M post-second booster (orange), pre-third booster (red, post-infection) and 1M post-third booster (yellow) vaccination time points. Neutralizing titers against all the isolates tested were found to be significantly higher at the 1M post-third booster vaccination time point when compared to the 1M post-second booster vaccination time point (*p*<0.01, Bonferroni-Dunn corrected multiple Wilcoxon t tests) and the pre-third booster vaccination time point (*p*<0.05, Bonferroni-Dunn corrected multiple Wilcoxon t tests). The pre-third booster vaccination time point was also found to have significantly higher neutralizing titers than the post-second booster vaccination time point for the omicron BA.1, BA.2 and BA.5 isolates (*p*<0.05, Bonferroni-Dunn corrected multiple Wilcoxon t tests). **(C)** Comparison of neutralizing titers against the different SARS-CoV-2 isolates between the naïve (blue) and hybrid (orange) groups at the post-second booster vaccination time point. **(D)** Comparison of neutralizing titers against the different SARS-CoV-2 isolates between the naïve (blue) and hybrid (orange) groups at the post-third booster vaccination time point. Neutralizing titers against the delta, omicron BA.1 and omicron BA.2 isolates were found to be significantly higher in the hybrid group when compared to the naïve group (*p*<0.05, Bonferroni-Dunn corrected multiple Mann-Whitney U tests). *p<0.05 and **p<0.01.

To assess neutralizing breadth in the mRNA vaccination groups with and without prior SARS-CoV-2 infection, 11 participants from the hybrid group were selected to match the 11 participants in the naïve group to allow a more direct comparison between the two groups. The participants from the hybrid group were selected based on matched age, sex, complete longitudinal follow up, and only having a detected SARS-CoV-2 infection between their post-second booster and pre-third booster vaccination time points (representing a time when the SARS-CoV-2 omicron BA.2 variant was dominant in Denmark). A summary of the 11 selected participants from the hybrid group can be found in **Supplementary Table 2**. Neutralizing breadth was measured against a panel of SARS-CoV-2 isolates representing delta and omicron (BA.1, BA.2, BA.5, BQ.1.1 and XBB.1.4) variants. Firstly, neutralizing breadth was compared at 1M post-second booster and 1M post-third booster vaccination time points within the naïve (**Figure 2A**) group and at 1M post-second booster, pre-third booster (post-infection) and 1M post-third booster vaccination time points in the hybrid group (**Figure 2B**). For the naïve group, when compared to the 1M post-second booster vaccination time point, significantly higher neutralizing titers were observed at the 1M post-third booster vaccination time point for the omicron BA.1, BQ.1.1 and XBB.1.4 isolates (*p*<0.05, Bonferroni-Dunn corrected Wilcoxon t tests). By contrast, in the hybrid group, when compared to the 1M post-second booster vaccination time point, neutralizing titers against all isolates were significantly higher at the 1M post-third booster vaccination time point (*p*<0.01, Bonferroni-Dunn corrected Wilcoxon t tests). Furthermore, at the pre-third booster vaccination time point, neutralizing titers were significantly higher against the omicron BA.1, BA.2 and BA.5 isolates when compared to the post-second booster vaccination time point (*p*<0.05, Bonferroni-Dunn corrected Wilcoxon t tests). When the pre-third booster and post-third booster vaccination time points were compared, significantly higher neutralizing titers were detected at the post-third booster vaccination time point against all tested SARS-CoV-2 isolates (*p*<0.05, Bonferroni-Dunn corrected Wilcoxon t tests). To see if the breadth of neutralization in the hybrid group was superior to the naïve group, neutralizing titers were compared between these two groups at 1M post-second booster vaccination (**Figure 2C**) and at 1M post-third booster vaccination (**Figure 2D**). At 1M post-second booster vaccination, no observable differences were seen between the naïve and hybrid groups. At the 1M post-third booster vaccination time point, although the hybrid group had noticeably higher neutralizing titers against all the isolates tested, significance was only detected against the delta, omicron BA.1 and omicron BA.2 isolates (*p*<0.05, Bonferroni-Dunn corrected Mann-Whitney U tests).

### 3.4 Detection of antigen-specific CD8^+^ T cell responses

**Figure 3:**
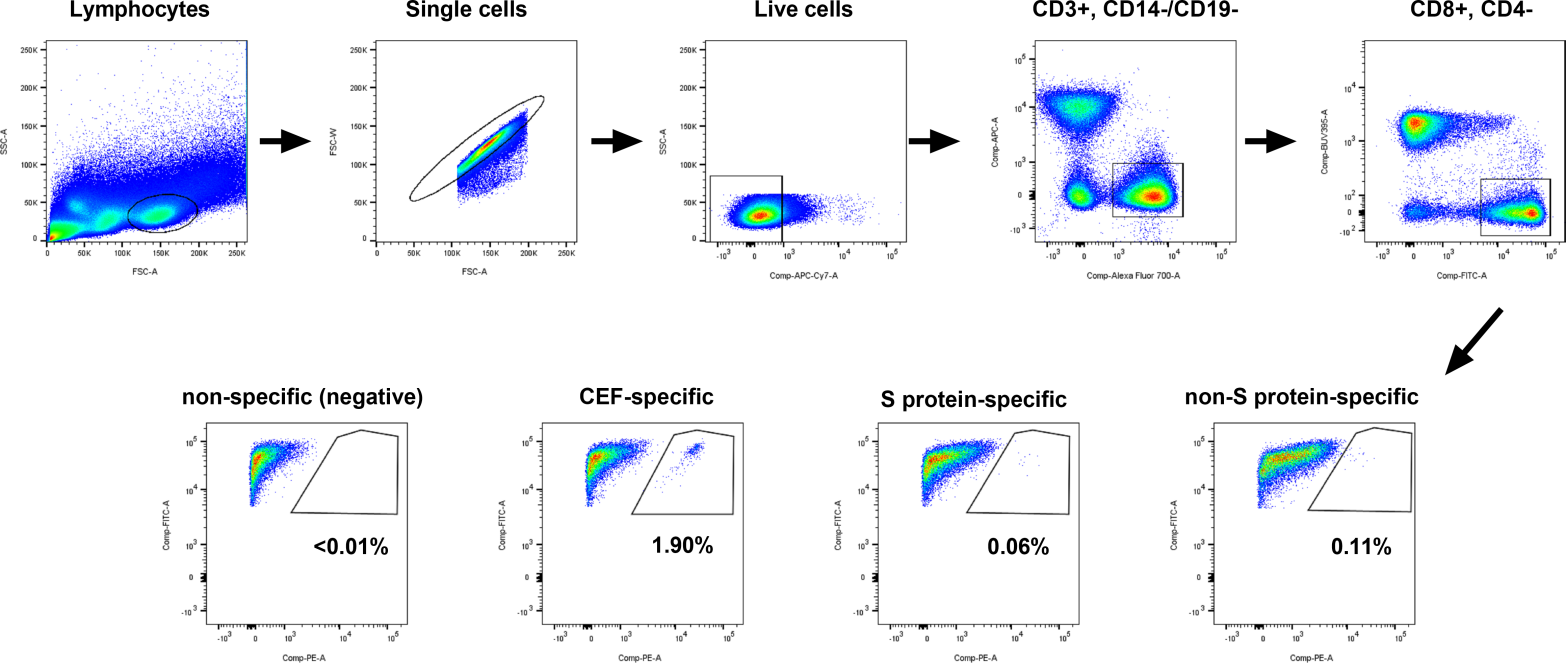
Gating strategy used for isolating antigen-specific CD8^+^ T cells from PBMCs. PBMCs were split into four wells. Each well of PBMCs contained Dextramer^®^ reagents specific to the individuals HLA allele(s), with the first well containing non-specific (negative control) Dextramer reagents, the second well containing CMV/EBV/Flu (CEF)-specific Dextramer reagents, the third well containing SARS-CoV-2 S protein-specific Dextramer reagents and the last well containing SARS-CoV-2 non-S protein-specific Dextramer reagents. Cells were analyzed on a BD Fortessa 5 laser instrument and the flow data were gated on lymphocytes, single cells, live cells (FVS780), CD3^+^ (AF700) and CD14^-^ (APC)/CD19^-^ (APC), CD8^+^ (FITC) and CD4^-^ (BUV395), and CD8^+^ (FITC) and Dextramer^®+^ (PE). The representative plots shown are for participant mRNA-11 at the post-third booster vaccination time point.

In order to identify SARS-CoV-2-specific CD8^+^ T cell responses, we selected S protein-specific and non-S protein-specific SARS-CoV-2 epitopes displayed by MHC on PE-labelled Dextramer reagents specific to HLA types A*01:01, A*02:01 and A*03:01 (**Supplementary Table 1**). Next, the 11 participants from the naïve group and the 11 matched participants from the hybrid group (i.e., the same 11 participants as studied above for cross-neutralizing antibodies) were HLA typed to identify those with suitable HLA A alleles. Of these participants, 10/11 of the naïve participants and 9/11 of the hybrid participants were found to have matching HLA A alleles (**Supplementary Table 3**). The MHC class I Dextramer reagents were used to identify SARS-CoV-2-specific CD8^+^ T cells in PBMCs isolated from peripheral blood at the same time points used in the neutralization analyses (**Figure 1A**). For each time point, analysis was done using four pools of Dextramer reagents, one containing negative control Dextramer reagents, one containing Dextramer reagents specific for other viral epitopes (CMV, EBV and Flu), one containing SARS-CoV-2 S protein-specific Dextramer reagents and one containing SARS-CoV-2 non-S protein-specific Dextramer reagents. Each pool of Dextramer reagents was analyzed separately from one another using flow cytometry and gated as shown in **Figure 3**. Any time points that had <5,000 CD8^+^ T cells were excluded from further analyses. In the naïve group, three showed consistently low levels of CD8^+^ T cells (<5,000 CD8^+^ T cells) and were excluded, meaning that only 7 subjects were included in the naïve group. A summary of the time points included and the detected antigen-specific CD8^+^ T cell frequencies can be found in **Supplementary Table 4**.

### 3.5 Assessment of S protein-specific CD8^+^ T cells

**Figure 4:**
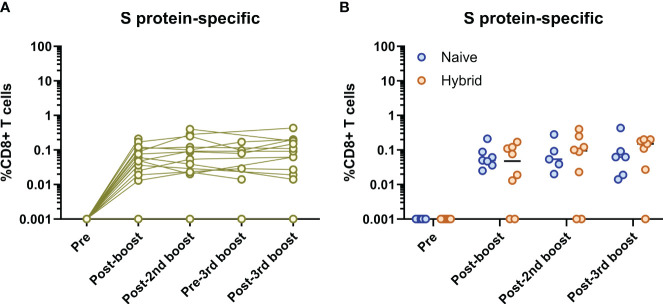
Analysis of SARS-CoV-2 S protein-specific CD8^+^ T cells. **(A)** Longitudinal frequencies of S protein-specific (yellow) CD8^+^ T cells in all individuals (n=16). No S protein-specific CD8^+^ T cells were detected at the pre-vaccination time point for all individuals and two individuals did not have any S protein-specific CD8^+^ T cells detected at follow-up time points. The remainder of individuals (n=14) showed continuous detection of S protein-specific CD8^+^ T cells at the post-booster vaccination time point and thereafter. No significant differences between each time point (excluding the pre-vaccination time point) were found (*p*>0.05, Dunn’s corrected Kruskal-Wallis test). **(B)** Comparison of S protein-specific CD8^+^ T cell frequencies between the naïve (blue) and hybrid (orange) groups. No significant differences were found at any time point (*p*>0.05, Holm-Sidak corrected multiple Mann-Whitney U tests). Time points from subjects were not included if<5,000 CD8^+^ T cells were detected.

Given that the vaccine formulas used in this study only contained the SARS-CoV-2 S protein, CD8^+^ T cell frequencies were first measured against epitopes specific to the SARS-CoV-2 S protein (**Figure 4**). For all individuals, S protein-specific CD8^+^ T cells were not detected at the pre-vaccination time point (**Figure 4A**). In two individuals, S protein-specific CD8^+^ T cells were not detected at any time point. For the remaining individuals, all follow-up time points showed continuously detectable S protein-specific CD8^+^ T cells at varying frequencies (**Figure 4A**). However, while some subjects showed a trend for increasing S protein-specific CD8^+^ T cell frequencies after each antigen exposure (i.e., infection or booster vaccination), no significant differences in the frequencies of S protein-specific CD8^+^ T cells were found at any time point (excluding comparisons to the pre-vaccination time point, *p*>0.05, Dunn’s corrected Kruskal-Wallis test). To see if there were any differences in the frequencies of S protein-specific CD8^+^ T cells between the naïve and hybrid groups, these groups were separated and compared at each time point (**Figure 4B**). Although slightly higher frequencies of S protein-specific CD8^+^ T cells were detected in the hybrid group at the post-third booster vaccination time point, no significant differences were detected at any time point (*p*>0.05, Holm Sidak corrected multiple Mann-Whitney U tests).

### 3.6 Assessment of non-S protein-specific and CEF-specific CD8^+^ T cells

**Figure 5:**
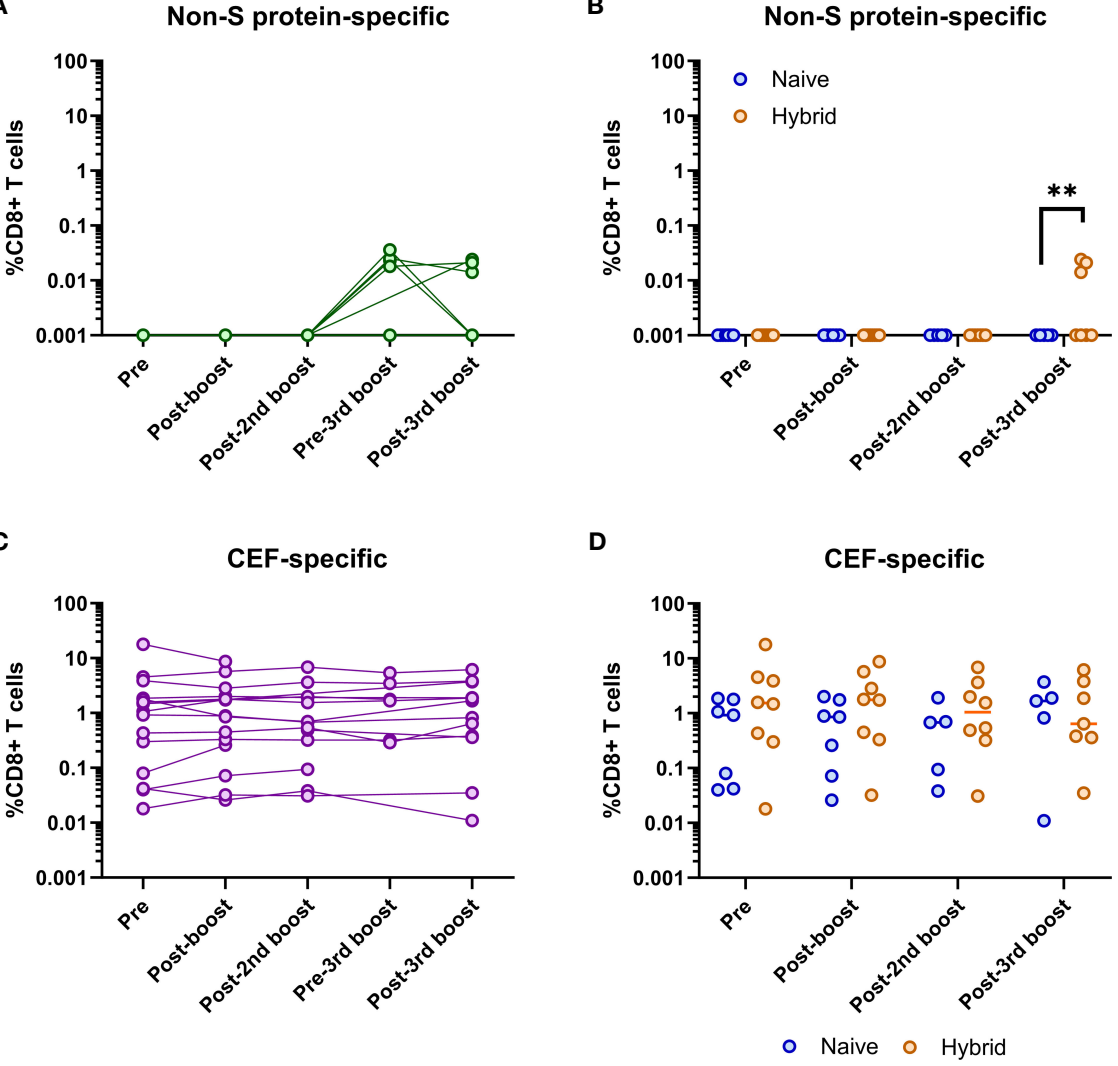
Analysis of SARS-CoV-2 non-S protein-specific and CEF-specific CD8^+^ T cell frequencies. **(A)** Longitudinal frequencies of non-S protein-specific (green) CD8^+^ T cells in all individuals (n=16). Detectable non-S protein-specific CD8^+^ T cells only occurred at the pre-third booster and post-third booster vaccination time points. No significant difference in these frequencies at these two time points was found (*p*>0.05, Mann-Whitney U test). **(B)** Comparison of non-S protein-specific CD8^+^ T cell frequencies between the naïve (blue) and hybrid (orange) groups. Non-S protein-specific CD8^+^ T cells were only found in the hybrid group. Significantly higher frequencies of non-S protein-specific CD8^+^ T cells were detected in the hybrid group at the post-third booster vaccination time point (*p*=0.005, Mann-Whitney U test). **(C)** Longitudinal frequencies of CMV/EBV/Flu (CEF)-specific (purple) CD8^+^ T cells in all individuals (n=16). No significant difference in these frequencies were found when all the time points were compared (*p*>0.05, Dunn’s corrected Kruskal-Wallis test). **(D)** Comparison of CEF-specific CD8^+^ T cell frequencies between the naïve (blue) and hybrid (orange) COVID-19 vaccinated groups. No significant differences were found in the frequencies of CEF-specific CD8^+^ T cells between the naïve and hybrid groups at any time point (Holm-Sidak corrected multiple Mann-Whitney U tests). Time points from subjects were not included if<5,000 CD8^+^ T cells were detected. **p<0.01.

Given that some of the individuals became infected, immunodominant epitopes outside of the S protein (i.e., non-S protein-specific epitopes) were assessed longitudinally (**Figure 5A**). Detection of non-S protein-specific CD8^+^ T cells only occurred at the pre-third booster vaccination and post-third booster vaccination time points (i.e., post-infection time points, **Figure 5A**). When these two time points were compared, no significant differences were found (*p*>0.05, Mann-Whitney U test). Detection of these CD8^+^ T cells only occurred in individuals in the hybrid group (**Figure 5B**). When compared at the post-third booster vaccination time point, the hybrid group had significantly higher non-S protein-specific CD8^+^ T cell frequencies than the naïve group (*p*=0.005, Mann-Whitney U test, **Figure 5B**). No other time points were compared as non-S protein-specific CD8^+^ T cells were not detected.

Longitudinal assessment of CEF-specific CD8^+^ T cells showed that they could be detected at all time points for all individuals at varying frequencies (**Figure 5C**). However, no significant differences were detected between any of the time points (*p*>0.05, Dunn’s corrected Kruskal-Wallis test). Similarly, when the frequencies of CEF-specific CD8^+^ T cells were compared between the naïve and hybrid groups, no significant differences were found (*p*>0.05, Holm-Sidak corrected multiple Mann-Whitney U tests, **Figure 5D**).

## 4 Discussion

Since the release of several COVID-19 vaccination strategies at the end of 2020, there has been a race between the emergence of SARS-CoV-2 variants and vaccination capable of protecting against evolving variants. With recent evidence showing that the newer BQ and XBB omicron variants are highly resistant to previously established neutralizing responses (5, 6), it’s important for vaccine formulas to be updated to help induce immunity that can protect against current circulating variants. While updated COVID-19 vaccine formulas have been shown to induce improved breadth of neutralization to emerging variants (5, 6), the level of neutralization is still significantly lower than that against the ancestral SARS-CoV-2 variant (Wuhan-Hu-1). However, continued antigen exposure, whether that be through vaccination or through infection, has shown improved breadth of neutralization to multiple SARS-CoV-2 variants (19). Similarly, the results presented in this study support the continued development of neutralizing breadth through additional antigen exposures, whether that be from additional vaccination, infection, or both. Particularly, individuals who had received the new Comirnaty^®^ bivalent vaccine (third booster vaccination) showed a large improvement in neutralization titers to the omicron variants tested. Although limited, other studies that have assessed the bivalent Spikevax^®^ vaccine (mRNA-1273.214) from Moderna have also shown comparable results to that seen in this study (27, 28). That is, the bivalent booster vaccination increased breadth of neutralization to omicron variants with a greater boost seen in those with prior infection. Interestingly, however, neutralization to the ancestral D614G isolate in this study, although boosted compared to pre-bivalent vaccination, was not brought back to comparable levels to that seen at post-second booster vaccination, unless individuals had had an infection (hybrid group). In a previous report assessing neutralization in individuals that received two booster vaccinations and individuals that received three booster vaccinations, while the monovalent third booster vaccination showed substantially increased neutralization titers against the ancestral D614G coronavirus pseudoparticle (CoVpp) compared to the bivalent third booster vaccination, the bivalent third booster vaccination showed much higher neutralization titers against the ancestral D614G CoVpp when compared to the neutralization titers after two booster vaccinations (29). However, this prior study did not use the same individuals for assessing neutralizing titers after three and four vaccine doses and failed to time-match the samples based on sample collection following vaccination, which has been shown to be important as neutralizing titers rapidly wane after 1M post-vaccination (19, 30). Therefore, this may explain the differences with the results found in the present study. This would make sense given that it is likely that memory B cells would be competing for the vaccine-induced antigens, and that having a lower amount of previously encountered antigen (Wuhan-Hu-1 S protein) would limit memory B cells from being activated, thus forcing differentiation and maturation of naïve B cells to the newly encountered omicron S antigens (BA.4/5 S protein). Therefore, compared to the third dose vaccination, which is exclusively the Wuhan-Hu-1 S protein, the bivalent vaccination would not induce as high neutralizing titers to the ancestral D614G isolate. However, given that the ancestral D614G SARS-CoV-2 variant is not circulating anymore, improvement of neutralizing titers against the newer BQ and XBB omicron variants is of higher importance. The data presented here, which is supported by others (6, 27, 30), shows that Comirnaty^®^ bivalent vaccination can indeed boost neutralization titers to the newer omicron variants. Although differences in monovalent and bivalent third booster vaccinations were not assessed in this study, others have shown that a bivalent third booster vaccination is superior at inducing improved neutralization titers against newer omicron variants (27, 31).

Although newer SARS-CoV-2 variants have been shown to be increasingly evasive to both infection- and vaccine-induced nAb responses, known CD8^+^ T cell epitopes have been found to be much more conserved between the different variants (11, 12). Given the apparent importance of CD8^+^ T cells in the complete clearance of SARS-CoV-2 infection (32, 33), antigen-specific CD8^+^ T cells were investigated in this study. Unlike previous studies assessing SARS-CoV-2-specific CD8^+^ T cell frequencies (13, 34, 35), this study compared the SARS-CoV-2-specific CD8^+^ T cell frequencies to the frequencies of other virus-specific CD8^+^ T cell responses (CEF pool). For SARS-CoV-2, the assay used here allowed tracking of the virus-specific T cell dynamics in response to both vaccination and infection. It was clear to see that, following prime-boost vaccination, S protein-specific CD8^+^ T cells could be detected and, following infection, detection of non-S protein-specific CD8^+^ T cells was observed. Importantly, these antigen-specific CD8^+^ T cells were not detected at pre-vaccination or at pre-infection time points, indicating that the CD8^+^ T cell epitopes employed in this study were specific to SARS-CoV-2 and that these epitopes were successful at identifying antigen-specific CD8^+^ T cells corresponding to the exposed antigen. When compared to other studies looking at SARS-CoV-2-specific CD8^+^ T cells, the frequencies found in this study are similar to that reported by others (13, 34, 35). Virus-specific T cell responses directed against CMV, EBV and Flu were detected at much higher frequencies compared to the SARS-CoV-2-specific CD8^+^ T cells, which could be due to several reasons. Firstly, CMV, EBV and Flu specific T cells were detected as one group, thus the CEF-specific cell numbers observed is the sum of responses to 3 viruses and not just one as for the SARS-CoV-2 responses measured. Secondly, these CD8^+^ T cells were likely formed from an infection with higher antigenic exposure than a vaccine, allowing greater expansion of antigen-specific CD8^+^ T cells prior to antigen clearance.

Interestingly, when the antigen-specific CD8^+^ T cell frequencies were examined longitudinally to either SARS-CoV-2 or CEF, no fluctuation in cell numbers was observed, even after additional antigen exposure (i.e., SARS-CoV-2 booster vaccination or infection). This is comparable to what has been observed in another study examining SARS-CoV-2-specific CD8^+^ T cells longitudinally (17). Given that it has been shown that the CD8^+^ T cell response to vaccinations peak around 10 days post-antigen exposure (36, 37), it is possible that frequencies of antigen-specific CD8^+^ T cells detected in this study, and by others (17), are after the antigen-specific CD8^+^ T cells have contracted back to a plateau, meaning that the detected antigen-specific CD8^+^ T cell frequencies in this study may have been past the expansion phase and, therefore, likely to be in a memory state. This would make sense considering SARS-CoV-2-specific memory CD8^+^ T cells have been shown to be long-lasting (38).

Although the epitopes used in this study were carefully selected based on previous reports of these epitopes being found at high rates among the HLA types selected for this study (12, 13), inclusion of additional epitopes may help improve detection frequencies of the SARS-CoV-2 non-S protein-specific CD8^+^ T cell frequencies. However, even epitopes like TTDPSFLGRY, which has been reported to be highly immunodominant in HLA A*01:01 individuals (13), were not found at high frequencies in A*01:01 individuals that acquired a SARS-CoV-2 infection in this study. However, this could be due to the participants having already established S protein-specific CD8^+^ T cell responses from COVID-19 vaccination, which will then be favored upon antigen re-exposure, especially considering the degree of conservation of CD8^+^ T cell epitopes between the SARS-CoV-2 variants. This phenomenon has been reported in individuals vaccinated against COVID-19 and suggested to be an effect of ‘immune imprinting’ (39). However, there is no evidence to suggest that immune imprinting induces better or worse disease outcomes after infection.

Overall, this study supports that the administration of a bivalent (Wuhan-Hu-1+BA.4/5 S mRNA) Comirnaty^®^ vaccination regime induces improved neutralizing antibody responses to omicron variants for both infection-naïve and infection-experienced individuals. Given the relationship between neutralizing titers and protection from severe COVID-19, the improved neutralizing responses seen from the bivalent Comirnaty^®^ booster vaccination are likely to improve protection efficacies. Furthermore, although SARS-CoV-2-specific CD8^+^ T cells were not found to be boosted following serial vaccinations, the number of specific T cells were sustained in those that had detectable responses. Lastly, there was no evidence of infection-experienced individuals having superior S protein-specific CD8^+^ T cell responses to infection-naïve vaccinated individuals, suggesting that infection-induced responses are not superior to vaccine-induced responses.

## Data availability statement

The original contributions presented in the study are included in the article/**Supplementary Material**. Further inquiries can be directed to the corresponding author.

## Ethics statement

The studies involving humans were approved by the Regional Ethical Committee (H-20025872) and Data Protection Agency (P-2020-357). The studies were conducted in accordance with the local legislation and institutional requirements. The participants provided their written informed consent to participate in this study.

## Author contributions

AU: Conceptualization, Data curation, Formal analysis, Funding acquisition, Investigation, Methodology, Software, Validation, Visualization, Writing – original draft, Writing – review & editing. CS: Funding acquisition, Resources, Writing – review & editing. KJ: Data curation, Formal analysis, Methodology, Resources, Validation, Writing – review & editing. AB: Investigation, Writing – review & editing. CF-A: Investigation, Writing – review & editing. LM: Investigation, Writing – review & editing. DI: Data curation, Methodology, Writing – review & editing. SLV: Resources, Writing – review & editing. JC: Formal analysis, Resources, Writing – review & editing, Investigation. MP: Resources, Writing – review & editing, Supervision. UF: Investigation, Methodology, Writing – review & editing. SR: Supervision, Writing – review & editing, Funding acquisition, Methodology, Resources. LB: Formal analysis, Resources, Supervision, Writing – review & editing. NW: Funding acquisition, Resources, Supervision, Writing – review & editing. JB: Conceptualization, Data curation, Formal analysis, Funding acquisition, Methodology, Project administration, Resources, Supervision, Validation, Writing – original draft, Writing – review & editing.

## References

[1] World Health Organization Coronavirus Disease (COVID-19) situation reports. Available at: https://www.who.int/emergencies/diseases/novel-coronavirus-2019/situation-reports/ (Accessed July, 2023).

[2] Kuodi P Gorelik Y Zayyad H Wertheim O Wiegler KB Abu Jabal K. Association between BNT162b2 vaccination and reported incidence of post-COVID-19 symptoms: cross-sectional study 2020-21, Israel. NPJ Vaccines. (2022) 7:101. doi: 10.1038/s41541-022-00526-5 36028498 PMC9411827

[3] Nguyen NN Houhamdi L Hoang VT Stoupan D Fournier PE Raoult D. High rate of reinfection with the SARS-CoV-2 Omicron variant. J Infect. (2022) 85:174–211. doi: 10.1016/j.jinf.2022.04.034 PMC9033627 35472367

[4] Perez-Alos L Armenteros JJA Madsen JR Hansen CB Jarlhelt I Hamm SR. Modeling of waning immunity after SARS-CoV-2 vaccination and influencing factors. Nat Commun. (2022) 13:1614. doi: 10.1038/s41467-022-29225-4 35347129 PMC8960902

[5] Kurhade C Zou J Xia H Liu M Chang HC Ren P. Low neutralization of SARS-CoV-2 Omicron BA.2.75.2, BQ.1.1 and XBB.1 by parental mRNA vaccine or a BA.5 bivalent booster. Nat Med. (2023) 29:344–7. doi: 10.1038/s41591-022-02162-x 36473500

[6] Miller J Hachmann NP Collier AY Lasrado N Mazurek CR Patio RC. Substantial neutralization escape by SARS-CoV-2 Omicron variants BQ.1.1 and XBB.1. N Engl J Med. (2023) 388:662–4. doi: 10.1056/NEJMc2214314 PMC9878581 36652339

[7] Touret F Giraud E Bourret J Donati F Tran-Rajau J Chiaravalli J. Enhanced neutralization escape to therapeutic monoclonal antibodies by SARS-CoV-2 omicron sub-lineages. iScience. (2023) 26:106413. doi: 10.1016/j.isci.2023.106413 36968074 PMC10015083

[8] Khoury DS Cromer D Reynaldi A Schlub TE Wheatley AK Juno JA. Neutralizing antibody levels are highly predictive of immune protection from symptomatic SARS-CoV-2 infection. Nat Med. (2021) 27:1205–11. doi: 10.1038/s41591-021-01377-8 34002089

[9] Cromer D Steain M Reynaldi A Schlub TE Wheatley AK Juno JA. Neutralising antibody titres as predictors of protection against SARS-CoV-2 variants and the impact of boosting: a meta-analysis. Lancet Microbe. (2022) 3:e52–61. doi: 10.1016/S2666-5247(21)00267-6 PMC8592563 34806056

[10] Brasu N Elia I Russo V Montacchiesi G Stabile SA De Intinis C. Memory CD8(+) T cell diversity and B cell responses correlate with protection against SARS-CoV-2 following mRNA vaccination. Nat Immunol. (2022) 23:1445–56. doi: 10.1038/s41590-022-01313-z 36138186

[11] Li H Chen Z Liu X Hu P. T cell epitopes are largely conserved in the SARS-CoV-2 Omicron subvariant (BA.1, BA.2, BA.3, and GKA). J Med Virol. (2022) 94:4591–2. doi: 10.1002/jmv.27925 PMC9348460 35676232

[12] Meyer S Blaas I Bollineni RC Delic-Sarac M Tran TT Knetter C. Prevalent and immunodominant CD8 T cell epitopes are conserved in SARS-CoV-2 variants. Cell Rep. (2023) 42:111995. doi: 10.1016/j.celrep.2023.111995 36656713 PMC9826989

[13] Saini SK Hersby DS Tamhane T Povlsen HR Amaya Hernandez SP Nielsen M. SARS-CoV-2 genome-wide T cell epitope mapping reveals immunodominance and substantial CD8(+) T cell activation in COVID-19 patients. Sci Immunol. (2021) 6:eabf7550. doi: 10.1126/sciimmunol.abf7550 33853928 PMC8139428

[14] Guerrera G Picozza M D'Orso S Placido R Pirronello M Verdiani A. BNT162b2 vaccination induces durable SARS-CoV-2-specific T cells with a stem cell memory phenotype. Sci Immunol. (2021) 6:eabl5344. doi: 10.1126/sciimmunol.abl5344 34726470

[15] Gluck V Grobecker S Kostler J Tydykov L Bertok M Weidlich T. Immunity after COVID-19 and vaccination: follow-up study over 1 year among medical personnel. Infection. (2022) 50:439–46. doi: 10.1007/s15010-021-01703-9 PMC8475821 34562263

[16] Richardson JR Gotz R Mayr V Lohse MJ Holthoff HP Ungerer M. SARS-CoV2 wild type and mutant specific humoral and T cell immunity is superior after vaccination than after natural infection. PLoS One. (2022) 17:e0266701. doi: 10.1371/journal.pone.0266701 35468147 PMC9037910

[17] Maringer Y Nelde A Schroeder SM Schuhmacher J Horber S Peter A. Durable spike-specific T cell responses after different COVID-19 vaccination regimens are not further enhanced by booster vaccination. Sci Immunol. (2022) 7:eadd3899. doi: 10.1126/sciimmunol.add3899 36318037 PMC9798886

[18] Solund C Underwood AP Fernandez-Antunez C Bollerup S Mikkelsen LS Villadsen SL. Analysis of Neutralization Titers against SARS-CoV-2 in Health-Care Workers Vaccinated with Prime-Boost mRNA-mRNA or Vector-mRNA COVID-19 Vaccines. Vaccines (Basel). (2022) 10:75. doi: 10.3390/vaccines10010075 35062736 PMC8780959

[19] Underwood AP Solund C Fernandez-Antunez C Villadsen SL Mikkelsen LS Fahnoe U. Durability and breadth of neutralisation following multiple antigen exposures to SARS-CoV-2 infection and/or COVID-19 vaccination. EBioMedicine. (2023) 89:104475. doi: 10.1016/j.ebiom.2023.104475 36870117 PMC9978324

[20] Underwood AP Solund C Fernandez-Antunez C Villadsen SL Winckelmann AA Bollerup S. Neutralisation titres against SARS-CoV-2 are sustained 6 months after onset of symptoms in individuals with mild COVID-19. EBioMedicine. (2021) 71:103519. doi: 10.1016/j.ebiom.2021.103519 34419923 PMC8375401

[21] Harris PA Taylor R Thielke R Payne J Gonzalez N Conde JG. Research electronic data capture (REDCap)–a metadata-driven methodology and workflow process for providing translational research informatics support. J BioMed Inform. (2009) 42:377–81. doi: 10.1016/j.jbi.2008.08.010 PMC2700030 18929686

[22] Ramirez S Fernandez-Antunez C Galli A Underwood A Pham LV Ryberg LA. Overcoming culture restriction for SARS-CoV-2 in human cells facilitates the screening of compounds inhibiting viral replication. Antimicrob Agents Chemother. (2021) 65:e00097–21. doi: 10.1128/AAC.00097-21 PMC8406809 33903110

[23] Iglewicz B Hoaglin D. Volume 16: how to detect and handle outliers, The ASQC basic references in quality control: statistical techniques. Mykytka EF Hoaglin D Ph. D., Editor; 1993

[24] Zhou Y Gammeltoft KA Ryberg LA Pham LV Tjornelund HD Binderup A. Nirmatrelvir-resistant SARS-CoV-2 variants with high fitness in an infectious cell culture system. Sci Adv. (2022) 8:eadd7197. doi: 10.1126/sciadv.add7197 36542720 PMC9770952

[25] Shiina T Suzuki S Ozaki Y Taira H Kikkawa E Shigenari A. Super high resolution for single molecule-sequence-based typing of classical HLA loci at the 8-digit level using next generation sequencers. Tissue Antigens. (2012) 80:305–16. doi: 10.1111/j.1399-0039.2012.01941.x 22861646

[26] Kim D Paggi JM Park C Bennett C Salzberg SL. Graph-based genome alignment and genotyping with HISAT2 and HISAT-genotype. Nat Biotechnol. (2019) 37:907–15. doi: 10.1038/s41587-019-0201-4 PMC7605509 31375807

[27] Chalkias S Harper C Vrbicky K Walsh SR Essink B Brosz A. A bivalent Omicron-containing booster vaccine against Covid-19. N Engl J Med. (2022) 387:1279–91. doi: 10.1056/NEJMoa2208343 PMC9511634 36112399

[28] Chalkias S Harper C Vrbicky K Walsh SR Essink B Brosz A. Three-month antibody persistence of a bivalent Omicron-containing booster vaccine against COVID-19. Nat Commun. (2023) 14:5125. doi: 10.1038/s41467-023-38892-w 37612300 PMC10447540

[29] Wang Q Bowen A Valdez R Gherasim C Gordon A Liu L. Antibody response to omicron BA.4-BA.5 bivalent booster. N Engl J Med. (2023) 388:567–9. doi: 10.1056/NEJMc2213907 PMC9847504 36630643

[30] Canetti M Barda N Gilboa M Indenbaum V Mandelboim M Gonen T. Immunogenicity and efficacy of fourth BNT162b2 and mRNA1273 COVID-19 vaccine doses; three months follow-up. Nat Commun. (2022) 13:7711. doi: 10.1038/s41467-022-35480-2 36513665 PMC9745767

[31] Zou J Kurhade C Patel S Kitchin N Tompkins K Cutler M. Neutralization of BA.4-BA.5, BA.4.6, BA.2.75.2, BQ.1.1, and XBB.1 with bivalent vaccine. N Engl J Med. (2023) 388:854–7. doi: 10.1056/NEJMc2214916 PMC9891359 36734885

[32] Liu J Yu J McMahan K Jacob-Dolan C He X Giffin V. CD8 T cells contribute to vaccine protection against SARS-CoV-2 in macaques. Sci Immunol. (2022) 7:eabq7647. doi: 10.1126/sciimmunol.abq7647 35943359 PMC9407944

[33] Niessl J Sekine T Buggert M. T cell immunity to SARS-CoV-2. Semin Immunol. (2021) 55:101505. doi: 10.1016/j.smim.2021.101505 34711489 PMC8529278

[34] Keeton R Tincho MB Ngomti A Baguma R Benede N Suzuki A. T cell responses to SARS-CoV-2 spike cross-recognize Omicron. Nature. (2022) 603:488–92. doi: 10.1038/s41586-022-04460-3 PMC8930768 35102311

[35] Schulien I Kemming J Oberhardt V Wild K Seidel LM Killmer S. Characterization of pre-existing and induced SARS-CoV-2-specific CD8(+) T cells. Nat Med. (2021) 27:78–85. doi: 10.1038/s41591-020-01143-2 33184509

[36] Knudsen ML Ljungberg K Kakoulidou M Kostic L Hallengard D Garcia-Arriaza J. Kinetic and phenotypic analysis of CD8+ T cell responses after priming with alphavirus replicons and homologous or heterologous booster immunizations. J Virol. (2014) 88:12438–51. doi: 10.1128/JVI.02223-14 PMC4248943 25122792

[37] Reinscheid M Luxenburger H Karl V Graeser A Giese S Ciminski K. COVID-19 mRNA booster vaccine induces transient CD8+ T effector cell responses while conserving the memory pool for subsequent reactivation. Nat Commun. (2022) 13:4631. doi: 10.1038/s41467-022-32324-x 35941157 PMC9358914

[38] Dan JM Mateus J Kato Y Hastie KM Yu ED Faliti CE. Immunological memory to SARS-CoV-2 assessed for up to 8 months after infection. Science. (2021) 371:eabf4063. doi: 10.1126/science.abf4063 33408181 PMC7919858

[39] Wang J Li K Mei X Cao J Zhong J Huang P. SARS-CoV-2 vaccination-infection pattern imprints and diversifies T cell differentiation and neutralizing response against Omicron subvariants. Cell Discovery. (2022) 8:136. doi: 10.1038/s41421-022-00501-3 36543767 PMC9769462

