# Supplementary material for: Neutralizing antibody and CD8+ T cell responses following BA.4/5 bivalent COVID-19 booster vaccination in adults with and without prior exposure to SARS-CoV-2
Source: Front Immunol. 2024 Mar 20;15:1353353. doi: 10.3389/fimmu.2024.1353353 (PMC10987722; doi:10.3389/fimmu.2024.1353353)
Supplement: Supplementary file 1 [file Table_1.docx]

Supplementary Material

**Supplementary Table 1. Summary of Dextramer® reagents used for identification of SARS-CoV-2-specific CD8^+^ T cells.**

| **SARS-CoV-2-specific Dextramer® reagents** | | |
| --- | --- | --- |
| **Allele** | **Epitope** | **Antigen** |
| A*0101 | LTDEMIAQY | Spike |
| A*0101 | WTAGAAAYY | Spike |
| A*0101 | FTSDYYQLY | ORF3a |
| A*0101 | TTDPSFLGRY | ORF1ab |
| A*0201 | YLQPRTFLL | Spike |
| A*0201 | FIAGLIAIV | Spike |
| A*0201 | ALWEIQQVV | ORF1ab |
| A*0201 | LLLDRLNQL | N |
| A*0201 | LLYDANYFL | ORF3a |
| A*0301 | KCYGVSPTK | Spike |
| A*0301 | GVYFASTEK | Spike |
| A*0301 | KTFPPTEPK | N |
| A*0301 | VVYRGTTTYK | ORF1ab |
| **CEF Dextramer® reagents** | | |
| A*0101 | VTEHDTLLY | CMV |
| A*0101 | CTELKLSDY | Influenza |
| A*0201 | NLVPMVATV | CMV |
| A*0201 | GILGFVFTL | Influenza |
| A*0201 | GLCTLVAML | EBV |
| A*0301 | KLGGALQAK | CMV |
| A*0301 | RLRAEAQVK | EBV |
| **Negative control Dextramer® reagents** | | |
| A*0101 | STEGGGLAY | non-specific |
| A*0201 | ALIAPVHAV | non-specific |
| A*0301 | GLFGAGAFK | non-specific |

**Supplementary Table 2. Summary of the 11 selected participants from the hybrid group.**

**Supplementary Table 3. Summary of HLA A types in the hybrid and naïve groups. HLA A*01:01:01 is represented as yellow, HLA A*02:01:01 is represented as green and HLA A*03:01:01 is represented as red.**

| **Group** | **Patient ID** | **Age (years)** | **Sex** | **Ethnicity** | **HLA type (A)** | |  |
| --- | --- | --- | --- | --- | --- | --- | --- |
|  |  |  |  |  |  |  |  |
| Hybrid | mRNA-001 | 75 | Male | Caucasian | A*01:01:01 | A*02:01:01 |  |
|  | mRNA-018 | 62 | Female | Caucasian | A*03:01:01 | A*02:01:01 |  |
|  | mRNA-024 | 49 | Female | Caucasian | A*23:01:01 | A*02:01:01 |  |
|  | mRNA-026 | 60 | Male | Caucasian | A*24:02:01 | |  |
|  | mRNA-027 | 48 | Female | Caucasian | A*03:01:01 | A*68:02:01 |  |
|  | mRNA-028 | 60 | Female | Caucasian | A*01:01:01 | A*30:04:01 |  |
|  | mRNA-035 | 53 | Male | Caucasian | A*01:01:01 | |  |
|  | mRNA-037 | 64 | Female | Caucasian | A*24:02:01 | A*29:02:01 |  |
|  | mRNA-038 | 52 | Female | Caucasian | A*31:01:02 | A*01:01:01 |  |
|  | mRNA-079 | 48 | Female | Caucasian | A*03:01:01 | A*02:01:01 |  |
|  | mRNA-098 | 32 | Male | Caucasian | A*01:01:01 | A*03:01:01 |  |
| Naïve | mRNA-005 | 41 | Female | Caucasian | A*25:01:01 | A*02:01:01 |  |
|  | mRNA-009 | 55 | Female | Caucasian | A*31:01:02 | A*02:01:01 |  |
|  | mRNA-011 | 48 | Female | Caucasian | A*01:01:01 | |  |
|  | mRNA-039 | 53 | Female | Caucasian | A*03:01:01 | A*24:02:01 |  |
|  | mRNA-047 | 73 | Male | Caucasian | A*25:01:01 | A*23:01:01 |  |
|  | mRNA-061 | 63 | Female | Caucasian | A*01:01:01 | A*02:01:01 |  |
|  | mRNA-073 | 50 | Female | Caucasian | A*02:01:01 | A*11:01:01 |  |
|  | mRNA-090 | 56 | Female | Caucasian | A*25:01:01 | A*02:01:01 |  |
|  | mRNA-097 | 52 | Female | Caucasian | A*23:01:01 | A*03:01:01 |  |
|  | mRNA-101 | 25 | Male | Caucasian | A*02:01:01 | A*03:01:01 |  |
|  | mRNA-102 | 58 | Female | Caucasian | A*25:01:01 | A*03:01:01 |  |

**Supplementary Table 4. Summary of antigen-specific CD8^+^ T cell frequencies detected as well as time point exclusion.** The grey boxes indicate unavailable time points. The red boxes indicate time points that had less than 5,000 CD8^+^ T cells.
